# Circulating Tumor DNA: Less Invasive, More Representative Method to Unveil the Genomic Landscape of Newly Diagnosed Multiple Myeloma Than Bone Marrow Aspirates

**DOI:** 10.3390/cancers14194914

**Published:** 2022-10-07

**Authors:** Yang Liu, Jiapei Guo, Yuting Yi, Xuan Gao, Lei Wen, Wenbing Duan, Zhaohong Wen, Yaoyao Liu, Yanfang Guan, Xuefeng Xia, Ling Ma, Rong Fu, Lihong Liu, Xiaojun Huang, Qing Ge, Jin Lu

**Affiliations:** 1 National Clinical Research Center for Hematologic Disease, Peking University Institute of Hematology, Peking University People’s Hospital, Beijing 100044, China; 2 NHC Key Laboratory of Medical Immunology, Department of Immunology, School of Basic Medical Sciences, Peking University, Beijing 100191, China; 3 Department of Computer Science and Technology, School of Electronic and Information Engineering, Xi’an Jiaotong University, Xi’an 710039, China; 4 Geneplus-Beijing Technology Limited, Beijing 102206, China; 5 State Key Laboratory of Microbial Resources, Institute of Microbiology, Chinese Academy of Sciences, Beijing 100101, China; 6 GenePlus-Shenzhen Clinical Laboratory, Shenzhen 518122, China; 7 Department of Hematology, Tianjin Medical University General Hospital, Tianjin 300052, China; 8 Department of Hematology, The Fourth Hospital of Hebei Medical University, Hebei 050011, China; 9 Department of Integration of Chinese and Western Medicine, School of Basic Medical Sciences, Peking University, Beijing 100191, China; 10 Collaborative Innovation Center of Hematology, Soochow University, Suzhou 215006, China

**Keywords:** circulating tumor DNA, multiple myeloma, prognosis

## Abstract

**Simple Summary:**

The study of malignant plasma cell DNA genomics in multiple myeloma is a hot topic, which is mainly based on one-site bone marrow aspirates. In this study, we showed that circulating tumor DNA targeted next-generation sequencing analysis revealed a more comprehensive genomic architecture than bone marrow aspirates in newly diagnosed multiple myeloma. Circulating tumor DNA mutation in the transcriptional regulation pathway and DNA repair pathway were independent predictors of progression-free survival. ctDNA alterations correlated with prognosis and therapy response in newly diagnosed multiple myeloma.

**Abstract:**

Multiple myeloma (MM) is highly heterogenous and dynamic in its genomic abnormalities. Capturing a representative image of these alterations is essential in understanding the molecular pathogenesis and progression of the disease but was limited by single-site invasive bone marrow (BM) biopsy-based genomics studies. We compared the mutational landscapes of circulating tumor DNA (ctDNA) and BM in 82 patients with newly diagnosed MM. A 413-gene panel was used in the sequencing. Our results showed that more than 70% of MM patients showed one or more genes with somatic mutations and at least half of the mutated genes were shared between ctDNA and BM samples. Compared to the BM samples, ctDNA exhibited more types of driver mutations in the shared driver genes, higher numbers of uniquely mutated genes and subclonal clusters, more translocation-associated mutations, and higher frequencies of mutated genes enriched in the transcriptional regulation pathway. Multivariate Cox analysis showed that age, ctDNA mutations in the transcriptional regulation pathway and DNA repair pathway were independent predictors of progression-free survival (PFS). Our results demonstrated sequencing of ctDNA provides more thorough information on the genomic instability and is a potential representative biomarker for risk stratification and in newly diagnosed MM than bone marrow.

## 1. Introduction

Multiple myeloma (MM) is a clonal plasma cell disease characterized by recurrent cytogenetic and molecular abnormalities including translocations, partial deletions, or monosomies of chromosomes 1, 13, and 17, chromosomal trisomies, and somatic mutations [1,2,3,4,5,6]. Although therapeutic advances prolonged the overall survival, the prognosis varied significantly due to the inter-patient and intra-clonal genetic heterogeneity of the disease. Several bone marrow (BM)-based genetic approaches such as detection of cytogenetic aberrations by fluorescence in situ hybridization (FISH), next-generation sequencing (NGS), and gene expression profiling by bulk or even single-cell RNA sequencing have been applied to evaluate the heterogeneity of MM [7,8]. In addition, the assessment of minimal/molecular residual disease (MRD) that has prognostic significance for MM also requires NGS or next-generation flow cytometry [9]. Although these approaches have been reported to recognize MM patient subgroups with high-risk features and unfavorable outcomes, they generally require an invasive sampling from a single BM site. The repetitive sampling, hemodilution of BM samples, patchy quality, tumor evolution-resulted spatial and temporal heterogeneity, and extramedullary disease all make BM-based evaluation challenging [10].

In cancer patients, circulating cell-free DNA (cfDNA) that contains tumor-derived DNA (ctDNA) has been believed to be superior to single-site tissue biopsy for diagnosis and longitudinal management due to its less invasiveness and better representation of up-to-date tumor genome abnormality, tumor genomic diversity, and spatial heterogeneity [11]. ctDNA is particularly attractive in the management of MM, which has a hallmark of a high level of cytogenetic heterogeneity during disease progression, between subclones in the same or different BM sites, and between BM and the extramedullary sites [5]. Indeed, the levels and genomic variations of ctDNA have been found to be correlated with high-risk groups, disease progression, and tumor burden of MM patients. It could also be used to monitor patient response to therapy [12,13,14,15,16,17]. Notably, the genetic profiling concordance between cf/ctDNA and BM aspirate samples in these studies was examined based on a small number of patients and the concordance rates ranged from 90.5% in 10 patients and ~94% in 13 patients, to 96% in 48 patients (only five genes were sequenced) [12,14,15]. It is thus important to perform a comparison of cf/ctDNA with matched BM samples in a larger cohort of MM patients to reexamine their concordance and their association with disease risk and disease progression. In addition, some mutations were specifically found in ctDNA but missed in BM samples [14]. Investigating these mutations uniquely present in ctDNA may facilitate a better understanding of the cytogenetic variations, their potential role in tumor progression, and their significance in MM management. To this end, paired samples from blood (for ctDNA), BM aspirates and oral swabs (SW) were collected from 82 patients with newly diagnosed MM (NDMM). We used the Enrich Rare Mutation Sequencing (ER-Seq) with a 413 gene panel (Supplementary Table S1) with coding and non-coding fusion regions to examine the genetic profiles of these samples. A detailed comparative study of gene mutations, enriched signaling pathways, and their prognostic significance between ctDNA and BM was performed. Thirty of these patients also had their samples re-collected after six rounds of chemotherapy to study the longitudinal changes within cytogenetic mutations and the relationship between mutations and therapeutic responses.

## 2. Materials and Methods

### 2.1. Patient Cohort and Sample Collection

This is a prospective observational cohort study (ChiCTR2000040795). Eighty-two patients with newly diagnosed multiple myeloma were enrolled at Peking University People’s Hospital from August 2018 to June 2019. There was no smoldering myeloma or plasma cell leukemia. The objectives of this study were to compare the circulating tumor DNA and bone marrow genomics and to explore the correlation between ctDNA genomics and clinical characteristics and therapeutic effect. All patients were treated according to the RVD/BCD regimen for induction therapy with some dose adjustments depending on the individual patient’s frailty score. All patients received novel agent-based treatment except for one early death after diagnosis. All patients provided written informed consent. Peripheral blood (PB) (10 mL), BM aspiration samples (5 mL) and paired oral swabs (SW) (0.6 mL) were sampled from each patient before treatment at the same time. Basic demographic and clinical information, including gender, age, biochemical indicators, and stage, were recorded. The study complied with the declaration of Helsinki and was approved by the Ethics Committee of this institution (Certificate 2019PHB180-01 and 2020PHB104-01).

### 2.2. Genomic DNA Extraction

Peripheral blood was collected in Streck tubes (Omaha, NE, USA). Plasma was separated within 4 h by centrifuging at 1600× *g* at 4 °C for 10 min. The supernatant was then centrifuged at 16,000× *g* at 4 °C for 10 min to remove the remaining cell debris. Oral swab DNA was extracted using prepIT-L2P ORAcollect DNA Extraction Kit (DNA Genotek Inc., Ottawa, ON, Canada), to be used as the germline control sample. Cell-free DNA was isolated from plasma using the QIAamp Circulating Nucleic Acid Kit (Qiagen, Hilden, Germany), and BM genomic DNA was extracted using a DNA extraction kit (Cwbiotech, China). DNA concentration was estimated using a Qubit fluorometer and a Qubit dsDNA HS analysis kit (Invitrogen, Waltham, MA, USA). cfDNA fragment length was assessed using an Agilent 2100 Bioanalyzer and the DNA HS kit (Agilent Technologies, Santa Clara, CA, USA).

### 2.3. Sequencing Library Preparation and Target Capture

Each genomic DNA preparation extracted from SW and BM aspiration samples was cut into 200–250-bp fragments using a Covaris S2 instrument (Woburn, MA, USA) before library construction. After end-repair and polyA tailing, adapters with unique identifiers were ligated to both ends of double-stranded cfDNA fragments. Indexed Illumina Next Generation Sequencing (NGS) libraries were prepared from cfDNA, BM tumor DNA and SW germline DNA using the KAPA DNA Library Preparation Kit (Kapa Biosystems, Wilmington, MA, USA) following the manufacturer’s protocol. Target capture was performed using custom SeqCap EZ libraries (Roche NimbleGen, Madison, WI, USA). In order to explore the comprehensive genetic characteristics of multiple myeloma, the capture probe was designed based on 1.7 Mb genomic regions of 413 genes, involved in the oncogenesis and progression of hematological malignancies, such as multiple myeloma, lymphoma, etc. (Table S1). Capture hybridization was performed according to the manufacturer’s recommendations. The captured DNA fragments were then amplified and pooled to generate several multiplexed libraries. Sequencing was performed on the Illumina HiSeq 3000 instrument with 75 × 75 paired-end reads according to the manufacturer’s recommendations using an Illumina TruSeq PE Cluster Generation Kit v3 and the TruSeq SBS Kit v3 (Illumina, San Diego, CA, USA).

### 2.4. Sequencing Data Analysis

Terminal adaptor sequences and low-quality reads (>50% N rate, >50% bases with Q < 5) were removed from the raw data of paired samples. Clean reads were mapped to the reference human genome (hg19) and aligned using Burrows–Wheeler Aligner (BWA, version 0.7.12-r1039) with default parameters [18]. Picard (version 1.98) was used to identify and mark the duplicates produced by polymerase chain reaction (PCR). Realignment and recalibration were performed using GATK (version 3.4–46-gbc02625). MuTect (version 1.1.4) [19], was used to detect somatic small insertions and deletions (InDels) and SNVs. Somatic copy number alterations were identified with CONTRA (version 2.0.8) [20]. According to informative single nucleotide polymorphisms (SNPs), an algorithm was used to identify loss of heterozygosity (LOH) [21]. Application of oligonucleotide arrays for coincident comparative genomic hybridization, ploidy status and loss of heterozygosity studies in human cancers. The adjusted variant allele frequency was calculated according to the following equation to adjust for stromal content and copy number, with tumor/stromal fractions and local copy number from CONTRA output [22]. Both analyses of LOH and variant allele frequency (VAF) adjusted were performed on the samples with 30% of tumor fractions.

*VAF adjusted = VAF observed × [1 + (2 × Stromal Fraction)/(Tumor Fraction × Local Copy Number)]*

For structural variation (SV), probes were designed to capture selected exons and introns based on previously detected SVs in genes. To identify SVs, in-house algorithms were developed to identify split-reads and discordant read-pairs. All final candidate variants were manually validated with the integrative genomics viewer browser.

### 2.5. Enrich Rare Mutation Sequencing (ER-Seq) of ctDNA

In order to confirm the accuracy of detection of somatic SNVs and InDels, which were of the low-alteration fraction in ctDNA, high-quality support reads with both forward and reverse strand read pairs were clustered by unique identifiers (UIDs) and filtered through the germline mutations, as well as screening for “background noise” false-positive ctDNA mutations. The ER-Seq tactics identify and remove errors generated by PCR or sequencing, and enable efficient and precise detection of rare mutations in ctDNA [23].

### 2.6. Clonal Population Structure Analysis

PyClone was used to analyze the clonal population structures in baseline ctDNA and paired BM samples from each patient [24]. The copy number information of each SNV was used as input. For each clustering process, a PyClone algorithm was run for 20,000 iterations with a burn-in of 2000 iterations using a beta-binomial model with the “total_copy_number” option [25]. The maximum VAF of somatic mutations in each sample was used as “Tumor content.” Other parameters were default. The cancer cell fraction (CCF) was inferred, and variants were clustered as previously described. Variants located in the cluster with the greatest mean CCF were defined as clonal and the rest were sub-clonal [24]. ctDNA level was analyzed as the average VAF of mutations in the clonal cluster. In each ctDNA sample, mTBI was analyzed using the mean VAF of clonal mutations.

### 2.7. Fluorescence In Situ Hybridization (FISH)

All patients’ FISH were performed after CD138 magnetic immunomagnetic sorting. In brief, mononuclear cells were obtained from 10 mL bone marrow aspirates by density gradient centrifugation, and 500 µL of CD138 magnetic beads were used for the sorting. The harvested cells were stored at −20 °C. All interphase FISH analyses were performed according to the manufacturer’s standard protocols, using the following probes (Peking GP Medical Technologies, Beijing, China): FITC-rhodamine-labelled unique sequence-specific break-apart rearrangement probes for the immunoglobulin heavy chain (IgH) locus at 14q32 (GLP IgH). If an IgH translocation was identified, dual-color and dual-fusion translocation probes were used to distinguish between t(4;14)(p16;q32), t(14;16)(q32;q23) and t(11;14)(q13;q32). Two hundred nuclei were analyzed for each probe and the cut-off points for positive values were established based on BM results from 20 healthy donors and that had 5% for the IgH rearrangement.

### 2.8. Statistical Analysis

Descriptive statistics, including the frequency (proportions) for categorical variables and the median (range, or IQR) for quantitative variables, were used to describe the patient demographic and clinical characteristics. Spearman’s correlation analysis was performed to estimate the correlation between different parameters. Categorical variables were compared using the χ2 test or Fisher’s exact test. Continuous variables were compared using a nonparametric test (Mann–Whitney U test). Variables with *p* values less than 0.2 according to univariate analysis were included and selected by a stepwise process to fit a multivariate regression model. SPSS 25.0 (SPSS Inc., Chicago, IL, USA), R version 4.2.0 (R Core Team, Vienna, Austria), and GraphPad Prism 8 (GraphPad Software Inc., San Diego, CA, USA) were used for the data analyses and graphing. 

## 3. Results

### 3.1. Higher Levels of Mutation Identified in ctDNA Than in BM

**Figure 1:**
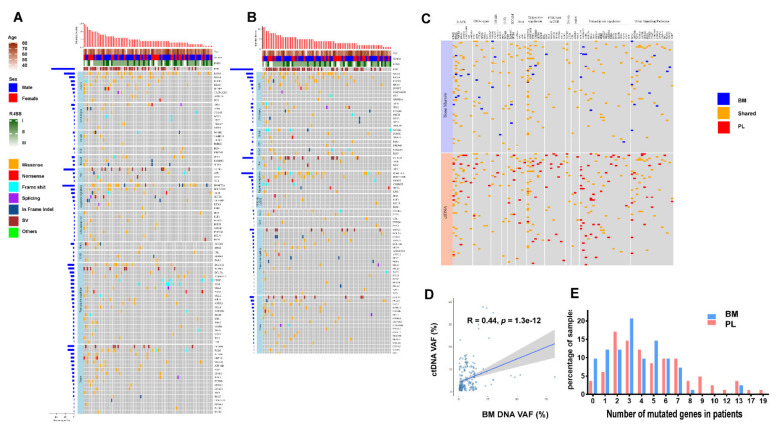
Comparison of the genomic landscape of ctDNA and Bone marrow (BM) DNA: (**A**) Somatic mutation profiles of newly diagnosed multiple myeloma (NDMM) patients from pre-treatment ctDNA sequencing of 413 cancer genes. Eighty-two patients were arranged along the *x*-axis. Age, sex, R-ISS stage, genes with somatic mutations were shown; (**B**) Somatic mutation profiles of NDMM patients from pre-treatment BM sequencing of 413 cancer genes; (**C**) Comparison of somatic mutation detected in ctDNA and paired BM DNA; (**D**) The relationship of variant allele frequencies (VAFs) between ctDNA and paired BM DNA; (**E**) Number of mutated genes in ctDNA and paired BM DNA.

The clinical characteristics of 82 NDMM patients were shown in Table 1. The sequencing of 82 NDMM patients with paired samples from ctDNA and BM both revealed vast inter-patient heterogeneity (Figure 1A–C, Supplementary Tables S2 and S3). The number of nonsynonymous nucleotide variant and/or insertion-deletion mutations (InDel) identified in ctDNA was 415 in total with an average of 5.1 mutations per sample. In matching BM samples, only 297 mutations were identified with an average of 3.6 mutations per sample (Supplementary Tables S2 and S3). Within 237 shared mutations obtained from ctDNA and BM (57.11% and 79.80%, respectively), we observed similar levels of overall variant allele frequencies (VAFs) (median 0.030 (IQR: 0.014, 0.084) and median 0.041 (IQR: 0.016, 0.105), respectively; *p =* 0.06) and a positive correlation in VAF (Spearman correlation coefficient r = 0.44, *p =* 1.3 × 10 ^−12^) between ctDNA and BM (Figure 1D) [12,14,15]. The number of unique mutations, however, was higher in ctDNA samples (178, 42.89%) than in the BM samples (60, 20.20%).

We further compared the number of mutated genes identified in paired samples. In ctDNA samples, 79 (96.34%) had one or more genes with somatic mutations. The median number was 4 (ranging from 0 to 19) and 18.29% of the patients had 8 or more mutated genes (Figure 1E). In the BM samples, however, 74 (90.24%) harbored somatic mutations with a median number of 3 (ranging from 0 to 13) mutated genes per sample and only 3.66% of the patients had 8 or more genes with mutation (Figure 1E). Notably, the patients with more than 14 mutated genes were only found in ctDNA (Figure 1E). Together, these data suggest that ctDNA represents a useful proxy for the mutational landscape analysis. Higher levels of mutations identified in ctDNA may further provide an advantage in identifying somatic mutations in patients with MM.

### 3.2. Unique Mutated Gene Profiles in ctDNA

We next studied the mutated gene profiles in MM patients. The most frequently mutated genes were shared between ctDNA and BM samples and most of them were putative myeloma driver genes, including *KRAS* (19.51% vs. 14.63%), *NRAS* (13.41% vs. 17.07%), *BRAF* (8.54% vs. 6.10%), *HIST1H1E* (9.76% vs. 9.76%), *TP53* (8.54% vs. 6.10%), *FGFR3* (6.10% vs. 7.32%), *DIS3* (7.32% vs. 6.10%) (Supplementary Tables S2 and S3). The prevalence of a large gene *PCLO* was also high, with 10.98% in ctDNA and 8.54% in the BM. Mutations in *PCLO* have been found in lymphoma, several types of solid tumors, and one report of myeloma [26,27]. However, its role in the pathogenesis of MM or other cancers remains unclear.

We then examined the mutational signatures of myeloma driver genes and found higher sensitivity of ctDNA in detecting genomic footprints [28]. For instance, the mutations in *KRAS* were found in 16 ctDNA and 12 BM samples (Supplementary Tables S2 and S3). Among them, two subjects (MM056 and MM068) that simultaneously harbored two driver mutations Q61H and G12x in their ctDNA did not have *KRAS* mutations detected in the BM. The patient MM061 that harbored two frequent driver mutations G12V, A146V and one less frequent driver mutation K117N in ctDNA only had A146V identified in the BM. The rare mutation L23R that has been reported in diffuse large B cell lymphoma but not in MM [29], was detected in ctDNA but not in the BM. The mutations in *BRAF* were found in seven ctDNA samples and five BM samples. The likely pathogenic variant G466A [30] was only identified in ctDNA but was missed in the BM sample.

We further investigated the mutated genes that were detected in one type of sample but were missed in another. Fifty genes with mutations were uniquely identified in ctDNA whereas only seven genes were uniquely found in the BM samples. The unique mutated genes that were detected in two or more patients were only observed in ctDNA, including *SF3B1* (4.88%), *RELN* (2.44%), *PTPN11* (2.44%), *MTOR* (2.44%), *FOXO3* (2.44%), *EP300* (2.44%), *CARD11* (2.44%), *BRD4* (2.44%), and *ARID1B* (2.44%) (Supplementary Tables S2 and S3). Notably, some of the patients with a high number of genes with the mutation also had more than one gene identified uniquely in ctDNA. For instance, *SF3B1* mutation occurred together with *EP300* mutation in MM009 patients (nine mutated genes in total) or with *CARD11* and *MTOR* mutations in MM029 (17 mutated genes in total). A similar pattern of unique mutations (*FOXO3, RELN* and *BRD4*) was segregated in MM068 (nine mutated genes in total with two hotspot variants in *KRAS*) (Supplementary Table S2). It indicates that these patients with unique mutations in ctDNAs have high levels of genomic instability. Notably, *SF3B1* mutation was mostly found in myelodysplastic syndromes (MDS), chronic lymphocytic leukemia, or MM with potential myeloid neoplasm [31]. The four MM patients with *SF3B1* mutation in our cohort showed a higher percentage of cells with PRAME expression, a cancer/testis antigen that is more heavily overexpressed in MDS [32,33]. The median levels of PRAME expression were 14.87% in the subjects with *SF3B1* mutation and 0.38% in those without. In addition, three out of four patients had 5–10% of cells with morphologic myelodysplasia. Whether these patients have a higher probability to develop myeloid neoplasm awaits further investigation.

All the mutated genes were then analyzed by over-representation analysis and 11 signaling pathways were enriched. The transcriptional regulation pathway (detected in 47 ctDNA and 26 BM samples out of 82 patient samples), MAPK signaling pathway (41 ctDNA and 37 BM samples), and epigenetic regulation pathway (37 ctDNA and 33 BM samples) were the most frequently mutated pathways (Figure 1A,B). As mentioned above, the mutated genes involved in the MAPK signaling pathway (*KRAS*, *NRAS*, *BRAF*, *FGFR3*) had similar prevalence in ctDNA and BM samples. In contrast, the prevalence of mutated genes in the transcriptional regulation pathway were generally higher in ctDNA than in the BM samples (Supplementary Tables S2 and S3), including *BCL11B* (10.98% in ctDNA vs. 3.66% in the BM), *MLL2* (3.66% vs. 1.22%)*, MLL3* (6.10% vs. 2.44%)*, MLL4* (3.66% vs. 2.44%), *ANKRD11* (6.10% vs. 2.44%)*, MAF* (6.10% vs. 2.44%)*, SPEN* (4.88% vs. 3.66%)*, ASXL1* (4.88% vs. 2.44%), *CHD3* (6.10% vs. 3.66%)*, BCL7A* (4.88% vs. 3.66%).

**Figure 2:**
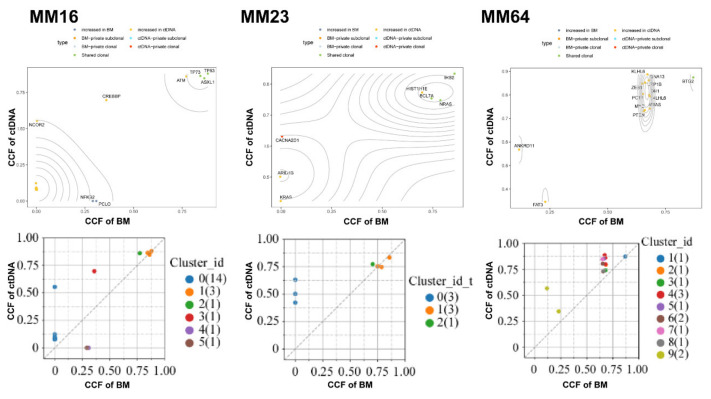
Representative genomic architecture derived from paired ctDNA versus BM DNA. Cancer Cell Fraction (CCF) of mutations was calculated in ctDNA and BM DNA. Each dot represents one mutation, and the color of each dot indicates the subclone that the given mutation was clustered to.

We also used PyClone to analyze the clonality of various mutations and tested if the subclonal composition in ctDNA is different from the matching BM samples. The cancer cell fraction (CCF) of each mutation was inferred in order to estimate the subclonal structure of the patients´ tumors. We observed a higher level of inter-patient clonal heterogeneity and identified higher numbers of subclonal clusters in ctDNA than in the BM samples. The median numbers of subclonal clusters were 3.2 (ranging from 0 to 11) in ctDNA and 2.2 (ranging from 0 to 9) in the BM. An example of the subclonal composition of three subjects (MM16, MM23, MM64) with founding clones carrying *TP53* or *NRAS* mutations was shown in Figure 2. Much higher CCF was observed in the founding clones in ctDNA than in the BM of these patients (Figure 2). Together, the results indicate that ctDNA sampling carries a more comprehensive mutation landscape with subclonal compositions when compared to BM sampling.

### 3.3. High Sensitivity of Structural Variations (SV) and Association of IGH Translocation with Gene Mutations in ctDNA

**Figure 3:**
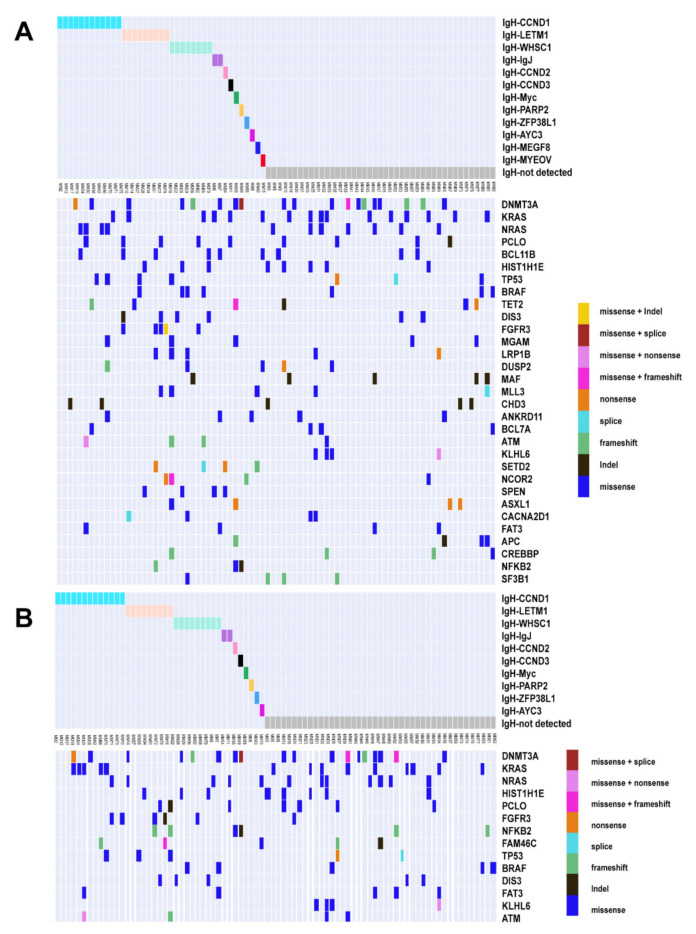
Genomic landscape under different IgH translocations. Somatic mutation profiles of NDMM patients grouping by IgH translocations. Different colors represent different IgH translocations and mutation types. (**A**) ctDNA; (**B**) BM.

We further investigated the structural variations (SV) and found that 39 patients (47.56%) in ctDNA and BM harbored *IGH*-associated translocation. As shown in Figure 3, the most prevalent *IGH*-associated translocations included *CCND1* (12 samples in ctDNA and 13 in BM), *WHSC1* (NSD2, eight in ctDNA and nine in BM), and previously unidentified *LETM1* (nine in ctDNA and nine in BM). We also found two *IGH*-associated chromosomal translocations in ctDNA but not in the BM, including *MEGF6* (MM40, 1.22%) and *MYEOV* (MM05, 1.22%). These two types of translocation were not reported previously in NDMM [34,35].

**Figure 4:**
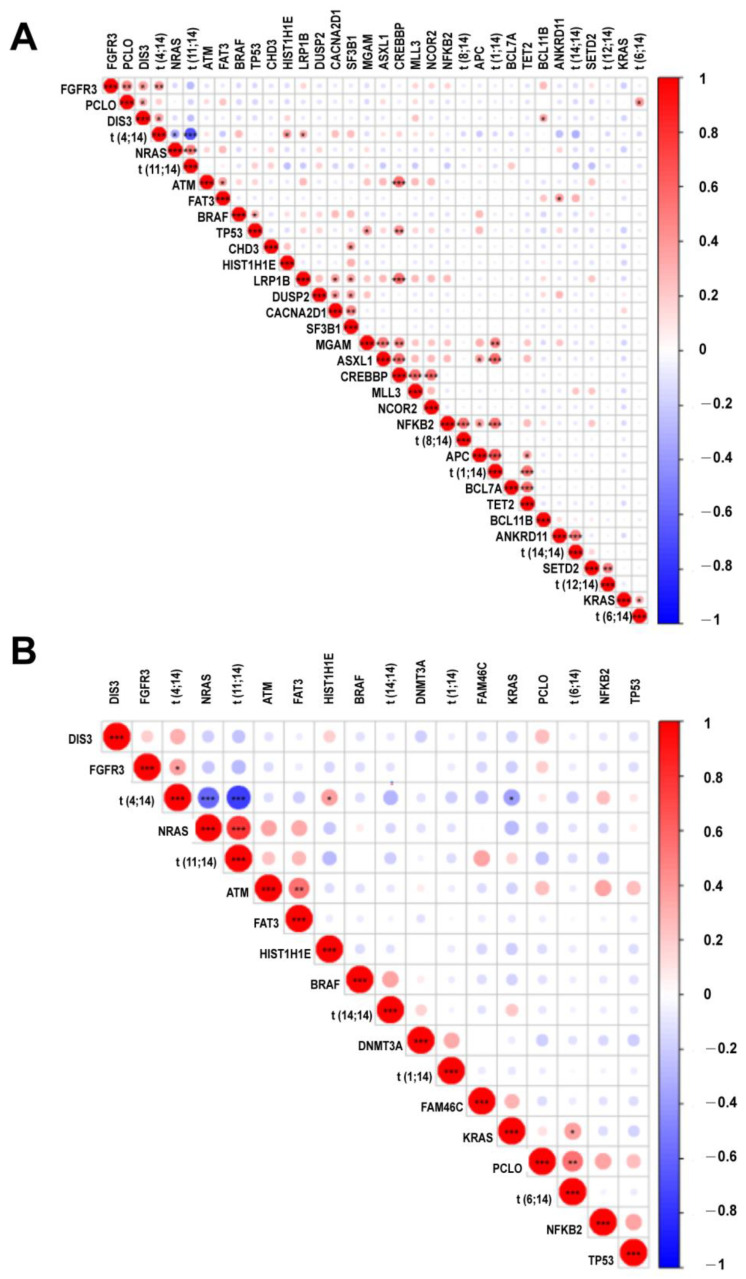
Correlation heatmap for hot somatic mutations and cytogenetic abnormalities. Correlation between mutations and recurrent cytogenetic abnormalities. Intensity of color shade represents the degree of correlation (blue, negative; red, positive) as per the scale. *, **, *** represent *p* value less than 0.05, 0.01, 0.001, respectively. (**A**) ctDNA; (**B**) BM.

We further calculated the correlation between translocations and mutations that were identified in more than 5% of patients (Figure 4). In ctDNA samples, mutations in *FGFR3, HIST1H1E, LRP1B,* and *DIS3* were positively correlated with t(4;14) while those in *NRAS* were negatively correlated with t(4;14). Positive correlations were also found between *NRAS* and t(11;14), *ANKRD11* and t(14;14), *KRAS/PCLO* and t(6;14), *NFKB2* and t(8;14). *SETD2* and t(12;14), and *APC/ASXL1/MGAM/NFKB2/TET2* and t(1;14). In the BM samples, however, much less associations were observed (Figure 4). As shown in Figure 4, a cosegregation of mutations in *MGAM*, *ASXL1,* and *CREBBP* was present in ctDNA but not in the BM samples.

We further compared the sequencing results with those obtained from FISH (with probes for *IGH* genes and three partner probes (t(4;14), t(11;14) and t(14;16)). In the patients with positive IgH translocation and partner probes (FISH), similar detection rates were found between ctDNA (28/38, 73.7%) and BM (30/38, 78.9%). In the patients that were positive in IgH translocation but negative in partner probes (FISH), 8/24 had their partner chromosomes identified by the SV analysis of ctDNA as well as BM. In the patients (21) that did not show positive signals detected by FISH, three were found harboring *IGH* translocation by ctDNA sequencing and one by BM sequencing. Together, these data suggest that ctDNA may have an advantage in identifying translocation-associated mutational subgroups.

### 3.4. Clinical Significance of the Molecular Tumor Burden Index in ctDNA

**Figure 5:**
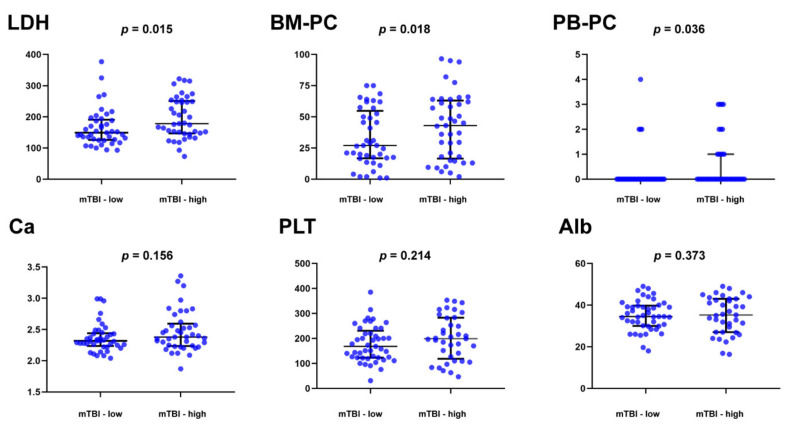
Clinical correlation of the molecular tumor burden index (mTBI) AND clinical parameters in 82 multiple myeloma patients. The patients were divided into mTBI-hi and mTBI-lo groups based on the median value. Compared to the MM patients in mTBI-lo group, those in mTBI-hi group had higher concentrations of serum lactate dehydrogenase (LDH) and higher percentages of bone plasma cells (BM-PC) and peripheral blood circulating plasma cells (PB-PC).

The molecular tumor burden index (mTBI) in ctDNA, evaluated by the mean VAF of the major clonal mutation, has a good correlation with tumor burden and has been used as a therapeutic response and prognostic biomarker in a number of solid tumors [36]. We thus examined the prognostic correlation of mTBI in MM. The patients were divided into mTBI^hi^ and mTBI^lo^ groups based on the median value. Compared to the MM patients in mTBI^lo^ group, those in mTBI^hi^ group had higher concentrations of serum LDH and higher percentages of BM plasma cells and circulating plasma cells (Figure 5). The levels of calcium, albumin, and platelets were not significantly different between the two groups (Figure 5). We also conducted a chi-square test for ranked data and found that the patients in mTBI^hi^ group had a more advanced ISS stage (*p* = 0.029), R-ISS stage (*p* = 0.012), and higher numbers of mutated genes in ctDNA (*p* = 0.005).

### 3.5. ctDNA as a Promising Parameter to Predict Inferior Survival

**Figure 6:**
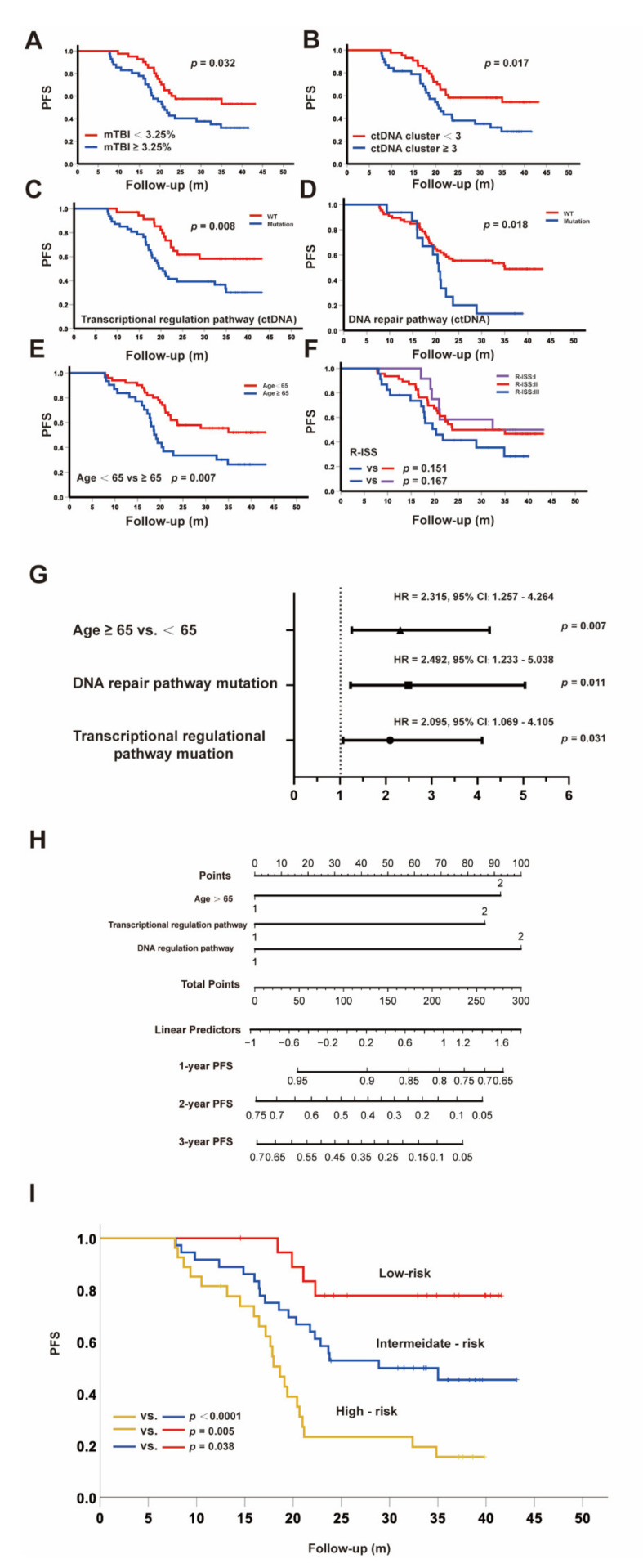
Multidimensional analysis of prognostic factors for PFS: (**A**–**F**) Kaplan–Meier curves for PFS according to the different factors; (**G**) Independent risk factors for PFS with different Hazard Ratios; (**H**) Nomogram models to predict PFS; (**I**) Kaplan–Meier curves for PFS according to risk factors.

We next used clinical data and several ctDNA parameters including mTBI, ctDNA cluster, and ctDNA mutation in pathways to determine their correlations with prognosis. The uni-variate Cox analysis showed that high-mTBI (≥median 3.25%) (HR: 1.901, 95%CI: 1.046−3.453, *p* = 0.035), high-ctDNA cluster (≥3) (HR: 2.025, 95%CI: 1.120−3.662, *p* = 0.020), DNA repair pathway mutation in ctDNA (HR: 2.152, 95%CI: 1.122−4.127, *p* = 0.021), Transcriptional regulation pathway mutation in ctDNA (HR: 2.297, 95%CI: 1.220−4.325, *p* = 0.010), Age ≥ 65 (HR: 2.207, 95%CI: 1.225−3.973, *p* = 0.008) were risk factors for PFS. Kaplan–Meier analysis of progression survival and log-rank tests were shown in Figure 6A–F.

Multivariate Cox regression analysis showed that Age ≥ 65 y, DNA repair pathway mutation in ctDNA, and transcriptional regulation pathway mutation in ctDNA were independent risk factors to predict PFS, as shown in Figure 6G. Moreover, we used ROC analysis to determine the best value for ctDNA mTBI was 2.765% (*p* = 0.015, sensitivity 68.9%, specificity 59.5%), and the uni- and multivariate Cox regression analysis for PFS was consistent with that grouping by the median.

The nomogram models were then formulated to predict PFS for NDMM patients based on the above independent risk factors (Figure 6H) [37,38]. The risk points were attributed according to the prognostic importance of these factors. Since the HR for these three risk factors were similar (Figure 6G), we categorized the entire cohort into three groups: low risk (no factors), intermediate risk (any one factor), and high risk (at least two factors). The PFS values among these groups were quite different (Figure 6I), with 2-year PFS rates of 77.8%, 52.8%, and 23.3% (*p* < 0.05) for low-, intermediate-, and high-risk groups, respectively. These results suggest that the nomogram with age and mutations in the transcriptional regulation pathway and DNA repair pathway is a reliable and quick approach to be used for risk stratification for PFS prediction.

### 3.6. Use of Clonal Composition Analysis of ctDNA to Monitor Therapeutic Response

**Figure 7:**
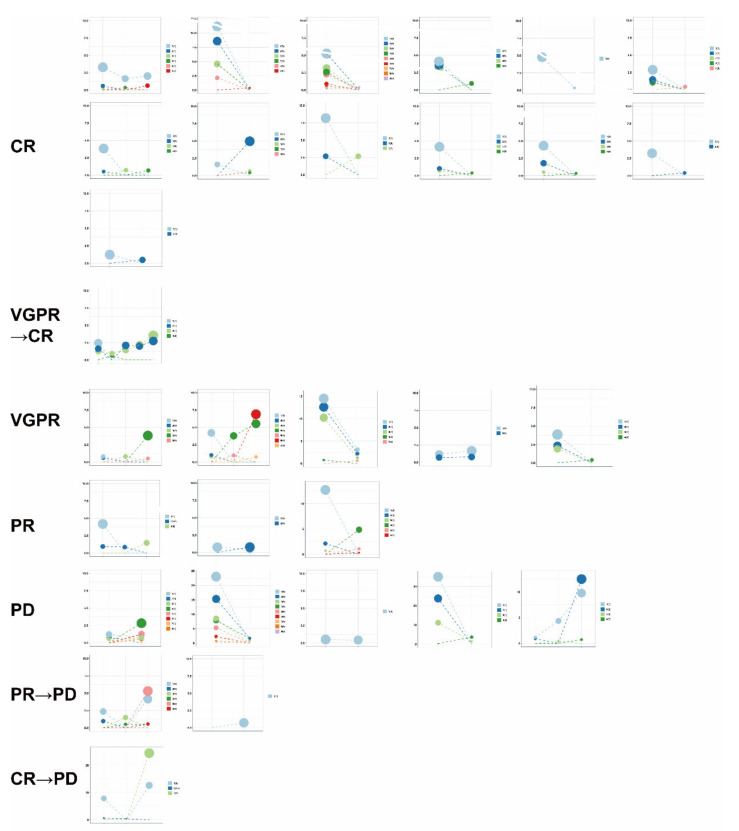
Clonal evolution before and after therapy according to different therapeutic responses. PyClone was employed to analyze the clonal structure using a Bayesian clustering method. For serial ctDNA, multiple inputs of each sample were used to analyze the serial clonal population. Each mutation’s CCF (cancer cell fraction) was calculated in ctDNA samples before and after therapy. The cluster with the highest CCF was identified as the clonal cluster, and mutations in this cluster were clonal mutations. Meanwhile, other clusters and mutations were considered subclonal. Each dot indicated one cluster, and a load of clusters was calculated with the mean VAF of each mutation that was clustered.

**Figure 8:**
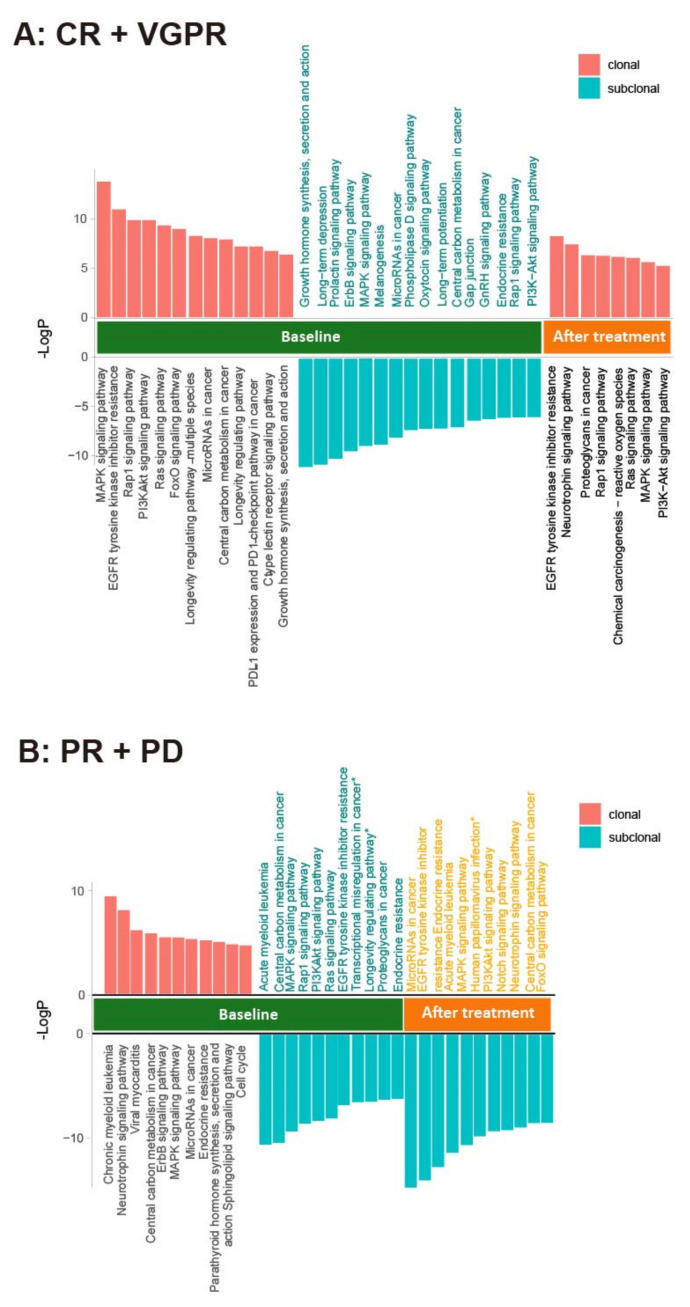
Involved pathways enriched in the clonal and subclonal mutations in ctDNA before and after treatment for (**A**) CR + VGPR; (**B**) PR + PD. PyClone was used to analyze the clonal population structures in baseline ctDNA and post-treatment ctDNA samples. The copy number information of each SNV was used as input. For each clustering process, a PyClone algorithm was run for 20,000 iterations with a burn-in of 2000, using a beta-binomial model with the “total_copy_number” option (Murtaza M, Dawson SJ, Pogrebniak K, Rueda OM, Provenzano E, Grant J et al. Multifocal clonal evolution characterized using circulating tumor DNA in a case of metastatic breast cancer. Nature communications. 2015; 6: 8760.) [25]. To clarify the functional role of clonal and subclonal genes, we performed pathway enrichment analysis in the mutations detected in before and after treatment samples.

To examine the association of ctDNA changes with therapeutic response, we re-sequenced the ctDNA of 30 patients after at least six cycles of treatment (Supplementary Table S4). The clonal evolution before and after therapy was calculated and a high level of heterogeneity was found in these patients (Figure 7). We further used GSEA to examine the pathways significantly enriched in the clonal and subclonal mutations. In the patients (20) with CR and VGPR, the post-treatment samples showed fewer pathways enriched in the clonal mutations and no pathways enriched in the subclonal mutations when compared to the pre-treatment samples (Figure 8A). In the patients (10) with PR and PD, however, the pathways enriched after treatment were only found in the subclonal mutations (Figure 8B). Notably, PDL1 expression and the PD1-checkpoint pathway in cancer were only enriched in pre-treated clonal mutations in CR and VGPR subjects. RAS and RAP1 signaling pathways were enriched in the clonal mutations in the patients with CR and VGPR but subclonal mutations in those with PR and PD. In addition, the clonal mutations in PR and PD subjects showed significant enrichment of cell cycle, implicating that these patients may gain additional benefit from chemotherapies that could induce cell cycle arrest. Together, these results suggest that the changes in the clonal composition of ctDNA have an advantage in monitoring therapeutic responses in patients.

## 4. Discussion

Multiple myeloma is a disease characterized by subclonal genetic abnormalities and a high degree of genetic heterogeneity within the tumor, between the patients, and induced by treatments. Although the ctDNA-based mutational genotyping is gaining adoption in the clinic for cancer surveillance, the detailed analysis and comparison of the mutational landscapes of ctDNA and BM in a relatively large cohort of patients with MM are still rare, hindering the wide application of the ctDNA-based approach in the monitoring of MM progression. In this study, we investigated the somatic aberrations in paired ctDNA and BM aspirates in 82 NDMM patients and wide genetic heterogeneity between patients and between pre- and post-treatment was observed. Consistent with previous reports [12,39], more than 70% of patients showed one or more genes with somatic mutations. We also found a good concordance of genetic profiles with more than 50% of the mutated genes shared between ctDNA and BM samples (with similar VAF), some of which were putative MM driver genes and some being enriched in the MAPK signaling pathway.

Importantly, our results further indicate that NGS of ctDNA provides a more comprehensive representation of mutational signatures of the myeloma genome. Compared to the BM samples, ctDNA exhibited more types of driver mutations in the shared driver genes, higher numbers of uniquely mutated genes and subclonal clusters, more translocation-associated mutations, and higher frequencies of mutated genes enriched in the transcriptional regulation pathway. The fact that more unique mutations were found in ctDNA than in BM agrees well with the findings by Mithraprabhu, S. et al. [16,17]. However, we used a much larger target capture panel (413) with coding and non-coding fusion regions in the measurement. A larger cohort (82) of patients with NDMM was also involved in the study. It provides a better image of the advantages of ctDNA sampling in the comprehensive evaluation of the genetic landscape of MM. In particular, multiple genes enriched in the transcriptional regulation pathway had higher mutation rates in ctDNA than in the BM. Although none of them have been proven to be putative MM driver genes, and some were even not reported in MM, the high prevalence of the mutated genes and their unique types of mutations warrant further investigation of their expression and roles in the pathogenesis of MM. For instance, the transcription factor BCL11B has been shown to be a tumor suppressor that is frequently mutated in lymphoid malignancies [40,41]. Neither the expression nor the function of BCL11B in myeloma cells is known. We found that 10.98% of ctDNA and 3.66% of BM samples had a single-site missense mutation in our patient cohort. Notably, a c.1395G > C mutation was seen in nine out of nine ctDNA and one out of three BM samples with mutated *BCL11B* (Supplementary Tables S2 and S3). Although the resulting p.E465D has not been reported before, its location in a region that encodes the critical DNA-binding zinc-finger domain (ZF3) of BCL11B suggests that the mutation may affect the folding of the zinc finger and/or DNA binding [42]. It is thus worth investigating the impact of p.E465D mutation on the function of BCL11B and on the pathogenesis of MM.

The mutations in myeloma often induce aberrant signaling and regulation. Consistent with previous reports [43,44,45], we also observed 48.8% of patients carrying mutations enriched in the MAPK pathway and 19.5% in the DNA repair pathway. However, our results showed less frequency of mutations enriched in the NF-κB pathway (9.8%) and a higher frequency of mutations in the PI3K pathway (18.3%) [3,46]. We further found that about 57.3% of patients had mutations enriched in the transcriptional regulation pathway, including *BCL11B* mentioned above. It suggests that a more in-depth investigation of the mutations in these genes and their effects on gene function is important in future research on the molecular mechanisms of MM.

When analyzing the association of mutation profiles with clinical measures, we further found a better correlation between ctDNA profile and clinical indicators. In particular, age, mutations in the transcriptional regulation pathway, and DNA repair pathway were indicated as independent predictors of PFS. To our knowledge, this is the first report in MM that uses the enriched pathways, instead of individual mutation or genes to predict the clinical outcome. The genetic profile of MM is highly heterogenous and even the mutation of the driver genes did not reach 20% in our 82-patient cohort. It is thus difficult to calculate the contribution of each mutation to prognosis. In addition, the deregulation of oncogenic pathways, but not mutations in a single gene determines the progression of MM [47]. Therefore, the use of mutated gene-enriched pathways obtained from ctDNA sequencing, including transcriptional regulation and DNA repair pathways that are critically involved in MM progression may increase the sensitivity of the approach and facilitate the risk stratification for PFS prediction.

In a course of treatment, the therapeutic pressure drives a dynamic alteration in the mutational burden, mutational profile, and structural aberrations. Compared to the invasive BM biopsies taken from a single-site, ctDNA may have an advantage in providing a representative image of clonal evolution as well as all high-risk clones that might present at other sites or focal lesions. The higher numbers of mutated genes and subclonal clusters found in ctDNA further enable us to examine the changes in pathways enriched in clonal and subclonal mutations in response to therapies. Interestingly, the patients with CR/VGPR and PR/PD showed different patterns after treatment, with the former having enriched pathways only in clonal mutations while the latter having enriched pathways only in subclonal mutations. Such a discrepancy indicates that subclonal mutations may play an important role in drug resistance and should be closely monitored during therapy.

Recently Luca Bertamini et al. analyzed 401 newly diagnosed MM patients and found that circulating tumor myeloma cells (CTC) higher than 0.07% represent inferior PFS (hazard ratio, 2.61; 95% CI, 1.49 to 2.97, *p* < 0.001) and OS (hazard ratio, 2.61; 95% CI, 1.49 to 4.56; *p* < 0.001) [48]. A detailed comparison study between ctDNA and CTC, especially the ctDNA mutation spectrum, is rare. These two methods have some similar advantages as liquid biopsies. The comparative study between these two methods needs further in-depth study and is useful to establish new more accurate risk stratification.

This study has some limitations. The follow-up period is less than 3 years, making it difficult to evaluate the association of the NGS results with the overall survival of the patients. The association of mutations/subclonal evolution with therapeutic responses remains to be validated due to the small number of patients that received pre- and post-therapy ctDNA sequencing in this study. A larger cohort may provide better information on longitudinal therapeutic monitoring. Furthermore, the BM DNA extraction was performed in all BM cells without plasma cell isolation, thus the low-frequency mutations may not have been detected because of the high number of other hematopoietic cells (progenitor and differentiated myeloid and lymphoid cells, and even erythroid precursors). However, from another point of view, ctDNA may also be partially derived from non-plasma cells. Therefore, the analysis of ctDNA, especially some common mutations from the myeloid cells, needs comprehensive analysis.

Taken together, we demonstrated the superiority of the ctDNA sequencing approach for comprehensive genetic profiling of MM and non-invasive real-time monitoring of patients receiving therapy. The results also indicate that mTBI and mutations identified from ctDNA-based NGS, in combination with other biomarkers, have a great potential to predict prognosis and therapeutic response in multiple myeloma.

## Figures and Tables

**Table 1:** Clinical characteristics of 82 NDMM patients.

Factors	Values
Sex, Male (%)	51 (62.2%)
Age (Median, Range)	62 (33–80)
DS (II/III)	1/4/77
ISS (I/II/III)	19/21/42
Renal insufficiency (SCr > 2 mg/dL)	21 (25.6%)
Low PLT (<100 × 10^9^/L)	10 (12.2%)
High LDH	18 (22.0%)
Del (17p)	10 (12.2%)
Gain/Amplification of 1q21	44 (53.7%)
Del (RB1)	38 (46.3%)
T(11;14)	20 (24.4%)
T(4;14)	18 (22.0%)
EMD	25 (30.5%)
Response (CR/VGPR/PR/NR or PD)	17/20/30/15

NDMM, Newly diagnosed multiple myeloma; DS, Durie Salmon stage; ISS, international staging system; SCr, Serum creatinine; PLT, platelet; LDH, Lactate dehydrogenase; Del, Deletion; EMD, extramedullary disease; CR, complete response; VGPR, very good partial response; PR, partial response; NR, no response; PD, progression of disease.

## Data Availability

All alterations detected in this study and de-identified patient clinical information was provided in the supplementary information. All other relevant data can be obtained from the corresponding authors of this study.

## Supplementary Materials

The following are available online at https://www.mdpi.com/article/10.3390/cancers14194914/s1, Supplementary Table S1. Gene list of 413-gene panel, Table S2: Supplementary Table S2. Somatic mutations detected in ctDNA in patients with NDMM, Supplementary Table S3. Bone marrow DNA mutations in patients with NDMM, Supplementary Table S4. Somatic mutations detected in post-treatment ctDNA in patients with MM.
